# Supplementary material for: Can systematic skewness factors predict future interest rates: Evidence from China
Source: PLoS One. 2026 Feb 5;21(2):e0334345. doi: 10.1371/journal.pone.0334345 (PMC12875505; doi:10.1371/journal.pone.0334345)
Supplement: Attachment — Submitted filename: EVALUATION PLOS One Article 2.docx [file pone.0334345.s003.docx]

# "Can Systematic Skewness Factors Predict Future Interest Rates: Evidence from China"

## MAJOR STRENGTHS

Novel Theoretical Contribution: The paper provides an important theoretical advancement in linking systematic skewness factors to macroeconomic expectations (that is interest rate measuring). This is a true gap in the literature since the majority of relevant literature focuses on behavioural explanations without exploring potential macroeconomic ties. The actual importance that investor's interest rate expectations manifest themselves in their preferences for stocks with systematic skewness is interesting and is consistent with economists' intuition.

Rigorous Empirical Design: There are several commendable features of the methodology. Using three systematic skewness measures (Cos, BM2, BHS) provides robustness. Including a wide-ranging set of control variables (both macroeconomic and classic pricing factors) shows a great deal of methodological sophistication. The heterogeneity analysis across bull and bear markets reveals economically interesting patterns that further our understanding of market manifestations.

Explicit Market Context: The paper focuses on China, an increasingly important financial system with its own unique institutional characteristics, thus contributing to our understanding of the international finance literature.

## Critical Weaknesses to Address

Theoretical Development: While the empirical findings are robust, the theoretical mechanism remains underdeveloped. The paper would benefit from a formal model or more rigorous theoretical framework explaining *why* systematic skewness factors should predict interest rates beyond the intuitive narrative. What is the equilibrium relationship? How do rational expectations reconcile with the documented patterns?

Endogeneity and Causality: The most significant methodological concern is potential reverse causality. Do systematic skewness factors predict interest rates, or do expectations about interest rates drive systematic skewness preferences? The paper treats predictive regressions as sufficient evidence, but instrumental variable approaches, Granger causality tests, or natural experiments would substantially strengthen causal claims. Current evidence demonstrates correlation, not causation.

Economic Magnitude: The paper reports statistical significance extensively but provides limited discussion of economic significance. What is the practical forecasting improvement over benchmark models? How do the predictive R² values compare to standard interest rate forecasting models? A comparison with AR models, Taylor rule specifications, or term structure models would contextualize the findings.

Sample Selection and Data Concerns: Excluding the smallest 30% of stocks by market capitalization to avoid "shell value" effects is pragmatic but potentially problematic. This could induce selection bias and limit generalizability. Additionally, the paper's treatment of the 1997-2024 sample period deserves scrutiny—this encompasses multiple regime changes in Chinese monetary policy and market regulation that warrant explicit discussion or subsample analysis.

Robustness Checks: The paper lacks several standard robustness tests including alternative forecasting horizons beyond 1-4 months, out-of-sample forecasting performance, and alternative specifications for constructing systematic skewness factors (e.g., different portfolio cutoffs than 30/30).

Readability and Presentation: While the paper is well-organized, several tables (especially Tables 3–6) are excessively dense. The addition of graphical summaries, conceptual diagrams, or flowcharts illustrating relationships between skewness, market sentiment, and interest rates would enhance readability and accessibility for a multidisciplinary audience.

Policy and Practical Implications: The conclusion section could more explicitly articulate the implications for monetary policy, portfolio management, and risk assessment. For instance, how might systematic skewness serve as a market-based indicator of future policy shifts or investor sentiment during liquidity tightening cycles?

**RECOMMENDATION**

**Accept with Major Corrections** addressing the theoretical framework, endogeneity concerns, and robustness checks. The core contribution is valuable, but these revisions are essential for publication.
