# Supplementary material for: Can systematic skewness factors predict future interest rates: Evidence from China
Source: PLoS One. 2026 Feb 5;21(2):e0334345. doi: 10.1371/journal.pone.0334345 (PMC12875505; doi:10.1371/journal.pone.0334345)
Supplement: Attachment — Submitted filename: Response to Reviewers.docx [file pone.0334345.s004.docx]

Dear Editor,

We sincerely appreciate your and all the reviewers’ critiques and valuable feedback. We are fortunate that our manuscript was eventually assigned to these reviewers, as their valuable feedback not only helped us improve the paper but also provided some insightful directions for future research. Please convey our heartfelt thanks to these experts.

Based on the feedback received, we have carefully revised the original manuscript. All changes are highlighted in blue text within the revised version. Below, you will find our point-by-point responses to the comments.

We hope the new version meets your standards, and if any shortcomings remain, please do not hesitate to point them out. We look forward to receiving any response regarding our submission and are pleased to address any further questions or comments you may have.

Xinyao Liang

Yichen Sun

**Response to comments on manuscript ID: PONE-D-25-52488**

(1) Your Comment:

***Theoretical Development:*** While the empirical findings are robust, the theoretical mechanism remains underdeveloped. The paper would benefit from a formal model or more rigorous theoretical framework explaining why systematic skewness factors should predict interest rates beyond the intuitive narrative. What is the equilibrium relationship? How do rational expectations reconcile with the documented patterns?

Our revision:

Thank you for this suggestion for revision. We completely agree with your perspective.

Investors’ hedging activities may trigger market liquidity tensions or asset sell-offs, increasing the vulnerability of the financial system. If such behavior exacerbates systemic risks, the central bank may adjust interest rates (for example, by lowering rates to provide liquidity) to smooth market volatility and maintain financial stability (Drechsler et al., 2021; Bocola and Lorenzoni, 2020).

Stocks exhibiting positive systematic skewness, which demonstrate the ability to generate positive returns during market downturns and thus meet investors’ hedging demands (Langlois, 2020; Baltussen et al., 2021), tend to be favored by investors when their hedging demands increase. This preference drives up the prices of these stocks, leading to lower future returns and consequently enhancing the value of the systematic skewness factor. Conversely, when investors’ hedging demands decline, the value of the systematic skewness factor also diminishes. Based on the above reasoning, we define the systematic skewness factors as the return difference between the 30% of stocks with the lowest (typically negative) systematic skewness and the 30% of stocks with the highest (typically positive) systematic skewness, and we employ numerical changes of systematic skewness factors to capture the time-varying nature of investors’ hedging demands.

To further elaborate on the theory, we construct a two-period model to illustrate the relationship between investors’ expectations of future interest rates and their hedging demands. This content has been added in Section 3 of the revised manuscript. For your convenience, the relevant section (pages 5~6 of the revised version) is quoted below:

**3. Theoretical Model Derivation**

We define the systematic skewness factors as the return difference between the 30% of stocks with the lowest (typically negative) systematic skewness and the 30% of stocks with the highest (typically positive) systematic skewness, and we employ numerical changes of systematic skewness factors to capture the time-varying nature of investors’ hedging demands. For instance, when investors’ hedging demands increase, they tend to favor stocks with positive systematic skewness, which drives up the prices of such stocks and leads to lower future returns, thereby resulting in an increase in the value of the systematic skewness factors. Conversely, when investors’ hedging demands decrease, it will reduce the value of the systematic skewness factors. Building upon this foundation, we will examine the predictive power of the systematic skewness factors on future interest rates.

To further elaborate on the theory, we construct a two-period model to illustrate the relationship between investors’ expectations of future interest rates and their hedging demands. We assume that investors have access to two types of assets - safe assets and risky assets, and that investors are risk-averse. When investors’ hedging demand increases, they tend to sell risky assets and seek refuge in safe assets. Concurrently, we assume that the central bank is forward-looking and that its monetary policy rule follows some variant of the Taylor rule, meaning its interest rate decisions respond to expectations about future output gaps and inflation. According to Ma et al. (2025), the reaction function of the Chinese monetary authorities must also account for exchange rate factors, in addition to the traditional Taylor rule framework. Therefore, we hypothesize that the reaction function of the Chinese monetary authorities follows the equation below:

$$r_{t+1}^{*}=r^{*}+E\left[ \pi_{t+1} \right]+\beta_{\pi}\left( E\left( \pi_{t+1} \right)-\pi^{*} \right)+\beta_{y}\left( E\left( y_{t+1} \right)-y^{*} \right)$$

$$+\beta_{e}\left( E\left( e_{t+1} \right)-e^{*} \right)+\varepsilon_{t+1}$$

where $r_{t+1}^{*}$ represents the desired future interest rate set by the Chinese monetary authorities based on current information, $r^{*}$ is the natural interest rate. $\pi_{t+1}$, $y_{t+1}$ and $e_{t+1}$ respectively denote the inflation level, economic output and exchange rate in period $t+1$, while $\pi^{*}$, $y^{*}$ and $e^{*}$ correspond to the central bank’s inflation target, economic output target and exchange rate target, respectively. Moreover, $\beta_{\pi}$, $\beta_{y}$ and $\beta_{e}$ are all positive.

According to Giglio et al. (2021), Atkinson et al. (2020) and Falato et al. (2021), investors’ hedging demands signal future economic recession, deflationary pressures and currency depreciation pressures. Therefore, our specified macroeconomic expectations equations are as follows:

$E\left( \pi_{t+1} \right)=f(\theta_{t})$, $\frac{\partial f(\theta_{t})}{\partial\theta_{t}}<0$

$E\left( y_{t+1} \right)=g(\theta_{t})$, $\frac{\partial g(\theta_{t})}{\partial\theta_{t}}<0$

$E\left( e_{t+1} \right)=h(\theta_{t})$, $\frac{\partial h(\theta_{t})}{\partial\theta_{t}}<0$

where $\theta_{t}$ represents investors’ hedging demands in period $t$. $f(\cdot)$、$g(\cdot)$、$h(\cdot)$ respectively represent the relationships between future expected inflation levels, economic output, exchange rates, and investors’ current hedging demands.

Therefore, the reaction function of the Chinese monetary authorities should follow the equation below:

$$r_{t+1}^{*}=r^{*}+f(\theta_{t})+\beta_{\pi}\left( f(\theta_{t})-\pi^{*} \right)+\beta_{y}\left( g(\theta_{t})-y^{*} \right)+\beta_{e}\left( h(\theta_{t})-e^{*} \right)$$

Since $f(\theta_{t})$、$g(\theta_{t})$ and $h(\theta_{t})$ are all decreasing functions of $\theta_{t}$, we can clearly observe that:

$$\frac{\partial r_{t+1}^{*}}{\partial\theta_{t}}=\left( 1+\beta_{\pi} \right)\frac{\partial f\left( \theta_{t} \right)}{\partial\theta_{t}}+\beta_{y}\frac{\partial g(\theta_{t})}{\partial\theta_{t}}+\beta_{e}\frac{\partial h(\theta_{t})}{\partial\theta_{t}}<0$$

From the above derivation, we can see that the future interest rate set by China’s monetary authorities is a decreasing function of investors’ current hedging demands. This implies that investors’ current hedging demands affect future interest rates by influencing the formulation of future monetary policy.

The derivation of the above theoretical model has been added in Section 3 of the revised manuscript (Pages 5~6 of the revised version) and is highlighted in blue text. **If there are any shortcomings in our revisions regarding this comment, please point them out, and we are willing to make further modifications.**

**References**

[1] Drechsler I, Savov A, Schnabl P. Banking on deposits: Maturity transformation without interest rate risk[J]. The Journal of Finance, 2021, 76(3): 1091-1143.

[2] Bocola L, Lorenzoni G. Financial crises, dollarization, and lending of last resort in open economies[J]. American Economic Review, 2020, 110(8): 2524-2557.

[3] Langlois H. Measuring skewness premia[J]. Journal of Financial Economics, 2020, 135(2): 399-424.

[4] Baltussen G, Da Z, Lammers S, Martens M. Hedging demand and market intraday momentum[J]. Journal of Financial Economics, 2021, 142(1): 377-403.

(2) Your Comment:

***Endogeneity and Causality:*** The most significant methodological concern is potential reverse causality. Do systematic skewness factors predict interest rates, or do expectations about interest rates drive systematic skewness preferences? The paper treats predictive regressions as sufficient evidence, but instrumental variable approaches, Granger causality tests, or natural experiments would substantially strengthen causal claims. Current evidence demonstrates correlation, not causation.

Our revision:

Thank you for this suggestion for revision. We completely agree with your perspective.

In Section 5.1 of the revised manuscript, we have supplemented Granger causality tests and instrumental variable tests to strengthen causal inference. For your convenience, the relevant content (Pages 11~13 of the revised version) is excerpted as follows:

**5.1 Endogeneity Tests**

This section includes Granger causality tests and instrumental variable tests.

**5.1.1 Granger Causality Tests**

Table 5 presents the Granger causality tests between the systematic skewness factors and future interest rates. It can be observed that the *Cos*, *BM2*, and *BHS* factors are Granger causes of both future short-term and long-term interest rates, while future short-term and long-term interest rates are not Granger causes of the *Cos*, *BM2*, and *BHS* factors. The results indicate that the systematic skewness factors have a unidirectional impact on both future short-term and long-term interest rates.

**Table 5**

Granger Causality Tests

| Panel A | Granger Causality Tests between Systematic Skewness Factors and Future Short-Term Interest Rates | | | |
| --- | --- | --- | --- | --- |
|  | Null Hypothesis | Obs | F-Statistic | Probability |
| (1) | *Cos* does not Granger Cause *Shibor* | 213 | 5.0030^***^ | 0.0075 |
| (2) | *Shibor* does not Granger Cause *Cos* | 213 | 0.6152 | 0.5415 |
| (3) | *BM2* does not Granger Cause *Shibor* | 213 | 5.1990^***^ | 0.0062 |
| (4) | *Shibor* does not Granger Cause *BM2* | 213 | 1.0914 | 0.3376 |
| (5) | *BHS* does not Granger Cause *Shibor* | 213 | 5.4801^***^ | 0.0048 |
| (6) | *Shibor* does not Granger Cause *BHS* | 213 | 0.9874 | 0.3742 |
| Panel B | Granger Causality Tests between Systematic Skewness Factors and Future Long-Term Interest Rates | | | |
|  | Null Hypothesis | Obs | F-Statistic | Probability |
| (7) | *Cos* does not Granger Cause *YTB* | 257 | 3.0128^*^ | 0.0509 |
| (8) | *YTB* does not Granger Cause *Cos* | 257 | 0.4260 | 0.6536 |
| (9) | *BM2* does not Granger Cause *YTB* | 257 | 3.5070^**^ | 0.0314 |
| (10) | *YTB* does not Granger Cause *BM2* | 257 | 0.5490 | 0.5782 |
| (11) | *BHS* does not Granger Cause *YTB* | 257 | 3.8434** | 0.0227 |
| (12) | *YTB* does not Granger Cause *BHS* | 257 | 0.7543 | 0.4714 |

**5.1.2 Instrumental Variable Tests**

We select the VIX index from the U.S. market as an instrumental variable for the systematic skewness factors. The VIX index reflects the future risk expectations of the core market in the global financial system, and its fluctuations quickly influence global market sentiment, including that of Chinese stock market, through channels such as capital flows, adjustments in multinational investor behavior, and contagion of risk preferences. As an external variable, the VIX index is not directly correlated with the internal fundamental factors of the Chinese stock market (such as domestic corporate earnings or policy changes), satisfying the exogeneity condition of an instrumental variable. Meanwhile, its systematic correlation with investor sentiment in the Chinese stock market ensures relevance.

Table 6 examines the predictive power of the VIX index on future interest rates. The results show that using the VIX index from the U.S. market as an instrumental variable for Chinese investor market sentiment reveals its significant predictive ability for short-term interest rates over the next five months and long-term interest rates over the next four months. This finding indicates that although the VIX index itself reflects volatility expectations in the U.S. stock market, it can influence fund flows driven by risk aversion sentiment and changes in policy expectations in Chinese stock market through the global financial sentiment transmission mechanism. Specifically, when the VIX index rises (indicating heightened global risk aversion), investors in the Chinese stock market may increase their purchases of stocks with positive systematic skewness due to elevated risk aversion, thereby affecting the value of the systematic skewness factors. When Chinese monetary authorities detect such market panic, they are likely to lower interest rates to maintain market stability. Conversely, a decline in the VIX index may drive interest rates upward. Therefore, a significant negative correlation exists between future interest rates and the current VIX index.

**Table 6**

Instrumental Variable Tests

| Panel A | ${Shibor}_{t}=\alpha+\beta_{1}{VIX}_{t-n}+\beta_{2}X_{t}+\varepsilon_{t}$ | | | | | |
| --- | --- | --- | --- | --- | --- | --- |
|  | $n=1$ | $n=2$ | $n=3$ | $n=4$ | $n=5$ | $n=6$ |
| *CONS* | 0.028^***^  (11.90) | 0.027^***^  (11.43) | 0.026^***^  (11.39) | 0.025^***^  (10.55) | 0.024^***^  (10.14) | 0.023^***^  (9.78) |
| $\beta_{VIX}$ | -3.50×10^-4***^  (-4.98) | -3.21×10^-4***^  (-4.29) | -3.26×10^-4***^  (-3.76) | -2.48×10^-4***^  (-2.82) | -2.01×10^-4**^  (-1.98) | -1.47×10^-4^  (-1.15) |
| *Macroeconomic Variables* | YES | YES | YES | YES | YES | YES |
| *Adj.R^2^* | 0.43 | 0.41 | 0.40 | 0.37 | 0.35 | 0.32 |
| Panel B | ${YTB}_{t}=\alpha+\beta_{1}{VIX}_{t-n}+\beta_{2}X_{t}+\varepsilon_{t}$ | | | | | |
|  | $n=1$ | $n=2$ | $n=3$ | $n=4$ | $n=5$ | $n=6$ |
| *CONS* | 0.035^***^  (19.96) | 0.035^***^  (20.05) | 0.035^***^  (20.36) | 0.035^***^  (20.79) | 0.036^***^  (21.35) | 0.037^***^  (22.16) |
| $\beta_{VIX}$ | -1.34×10^-4***^  (-2.86) | -1.20×10^-4**^  (-2.49) | -1.17×10^-4**^  (-2.25) | -1.04×10^-4*^  (-1.95) | -0.75×10^-4^  (-1.43) | -0.80×10^-4^  (-1.50) |
| *Macroeconomic Variables* | YES | YES | YES | YES | YES | YES |
| *Adj.R^2^* | 0.32 | 0.32 | 0.31 | 0.29 | 0.27 | 0.28 |

*Note:* The significance levels are adjusted using the Newey-West method, with the lag length selected as specified $T^{0.25}\approx4$. ***, ** and * denote significance at the 1%, 5%, and 10% levels, respectively. The values in parentheses are Newey-West adjusted t values.

The above content has been added in Section 5.1 of the revised manuscript (Pages 5~6 of the revised version) and is highlighted in blue text. **If there are any shortcomings in our revisions regarding this comment, please point them out, and we are willing to make further modifications.**

(3) Your Comment:

***Economic Magnitude:*** The paper reports statistical significance extensively but provides limited discussion of economic significance. What is the practical forecasting improvement over benchmark models? How do the predictive R^2^ values compare to standard interest rate forecasting models? A comparison with AR models, Taylor rule specifications, or term structure models would contextualize the findings.

Our revision:

Thank you for this suggestion for revision. We completely agree with your perspective.

In Section 5.3 of the revised manuscript, we have added out-of-sample forecasting and comparisons with benchmark models. For your convenience, the relevant content (Page 17 of the revised version) is quoted below:

**5.3 Out-of-Sample Forecasting and Comparisons with Benchmark Models**

This section fits the model using historical data from the past five years and forecasts monthly interest rates for the next twelve months. The prediction errors are measured using MSE, RMSE and MAE, with the results shown in Table 9. Whether predicting short-term or long-term interest rates, the prediction errors based on the systematic skewness factors are significantly smaller than those based on the AR model and the extended Taylor rule model proposed by Ma et al. (2025).

**Table 9**

Out-of-Sample Forecasting and Comparisons with AR Model and Extended Taylor Rule Model by Ma et al. (2025)

| Panel A | Out-of-Sample Forecasting of Short-Term Interest Rates | | |
| --- | --- | --- | --- |
| Models | MSE | RMSE | MAE |
| Predicting Shibor Based on *Cos* Factor | 3.15×10^-6^ | 0.0018 | 0.0015 |
| Predicting Shibor Based on *BM2* Factor | 2.89×10^-6^ | 0.0017 | 0.0013 |
| Predicting Shibor Based on *BHS* Factor | 2.34×10^-6^ | 0.0015 | 0.0011 |
| Predicting Shibor Based on AR Model | 4.88×10^-6^ | 0.0028 | 0.0023 |
| Predicting Shibor Based on Extended Taylor Rule Model by Ma et al. (2025) | 7.45×10^-6^ | 0.0047 | 0.0034 |
| Panel B | Out-of-Sample Forecasting of Long-Term Interest Rates | | |
| Models | MSE | RMSE | MAE |
| Predicting YTB Based on *Cos* Factor | 7.13×10^-6^ | 0.0027 | 0.0023 |
| Predicting YTB Based on *BM2* Factor | 6.87×10^-6^ | 0.0026 | 0.0022 |
| Predicting YTB Based on *BHS* Factor | 6.42×10^-6^ | 0.0025 | 0.0021 |
| Predicting YTB Based on AR Model | 7.75×10^-6^ | 0.0028 | 0.0025 |
| Predicting YTB Based on Extended Taylor Rule Model by Ma et al. (2025) | 10.72×10^-6^ | 0.0041 | 0.0035 |

The above content has been added in Section 5.3 of the revised manuscript (Page 17 of the revised version) and is highlighted in blue text. **If there are any shortcomings in our revisions regarding this comment, please point them out, and we are willing to make further modifications.**

(4) Your Comment:

***Sample Selection and Data Concerns:*** Excluding the smallest 30% of stocks by market capitalization to avoid “shell value” effects is pragmatic but potentially problematic. This could induce selection bias and limit generalizability. Additionally, the paper’s treatment of the 1997-2024 sample period deserves scrutiny - this encompasses multiple regime changes in Chinese monetary policy and market regulation that warrant explicit discussion or subsample analysis.

Our revision:

Thank you for this suggestion for revision. We completely agree with your perspective.

In the revised manuscript, we have adjusted the data sample by no longer excluding the smallest 30% of stocks by market capitalization and have re-reported all empirical results accordingly. **These modifications are reflected throughout the empirical findings and are highlighted in blue text.**

Additionally, in Section 5.2, we divided the sample into bull-market and bear-market phases and separately reported the empirical results for these two subsample tests. Moreover, we also used the 2005 equity division reform in Chinese stock market as a breakpoint, dividing the sample into pre-2005 and post-2005 periods. The empirical results similarly support the research conclusions of this paper. However, given the already substantial length of the manuscript, these findings were not included in the revised version. If required, we would be glad to supplement this part of the empirical results into the revised manuscript.

For your convenience, the relevant content (Pages 13~17 of the revised version) is excerpted as follows:

**5.2 Subsample Tests**

Based on the modified BB turning point method (Pagan and Sossounov, 2003), the Chinese stock market’s bull and bear market cycles are divided into periods, as shown in Figure 2. We can find that from January 2000 to December 2024, the Chinese stock market can be divided into six bull and bear cycles, with a cumulative bull market duration of 132 months and a cumulative bear market duration of 168 months.

**Figure 2**

The division of bull and bear markets in the Chinese stock market

*Note:* Figure 2 shows the Shanghai Composite Index points from January 2000 to December 2024. The shaded areas represent bull market periods, while the unshaded areas represent bear market periods.

|  | 是否为牛市 | 上证综合指数 |
| --- | --- | --- |
| 2000-01-04 | 7000 | 1406.37 |
| 2000-01-05 | 7000 | 1409.68 |
| 2000-01-06 | 7000 | 1463.94 |
| 2000-01-07 | 7000 | 1516.6 |
| 2000-01-10 | 7000 | 1545.11 |
| 2000-01-11 | 7000 | 1479.78 |
| 2000-01-12 | 7000 | 1438.02 |
| 2000-01-13 | 7000 | 1424.44 |
| 2000-01-14 | 7000 | 1408.85 |
| 2000-01-17 | 7000 | 1433.33 |
| 2000-01-18 | 7000 | 1426.62 |
| 2000-01-19 | 7000 | 1440.72 |
| 2000-01-20 | 7000 | 1466.86 |
| 2000-01-21 | 7000 | 1465.09 |
| 2000-01-24 | 7000 | 1477.34 |
| 2000-01-25 | 7000 | 1476.51 |
| 2000-01-26 | 7000 | 1481.12 |
| 2000-01-27 | 7000 | 1506.77 |
| 2000-01-28 | 7000 | 1535 |
| 2000-02-14 | 7000 | 1673.94 |
| 2000-02-15 | 7000 | 1670.67 |
| 2000-02-16 | 7000 | 1693.11 |
| 2000-02-17 | 7000 | 1640.65 |
| 2000-02-18 | 7000 | 1668.09 |
| 2000-02-21 | 7000 | 1677.14 |
| 2000-02-22 | 7000 | 1632.29 |
| 2000-02-23 | 7000 | 1594.93 |
| 2000-02-24 | 7000 | 1634.31 |
| 2000-02-25 | 7000 | 1631.58 |
| 2000-02-28 | 7000 | 1704.86 |
| 2000-02-29 | 7000 | 1714.58 |
| 2000-03-01 | 7000 | 1697.74 |
| 2000-03-02 | 7000 | 1713.01 |
| 2000-03-03 | 7000 | 1738.02 |
| 2000-03-06 | 7000 | 1681.09 |
| 2000-03-07 | 7000 | 1694.8 |
| 2000-03-08 | 7000 | 1726.03 |
| 2000-03-09 | 7000 | 1732.35 |
| 2000-03-10 | 7000 | 1705.05 |
| 2000-03-13 | 7000 | 1728.88 |
| 2000-03-14 | 7000 | 1685.35 |
| 2000-03-15 | 7000 | 1681.47 |
| 2000-03-16 | 7000 | 1607.53 |
| 2000-03-17 | 7000 | 1658.6 |
| 2000-03-20 | 7000 | 1699.25 |
| 2000-03-21 | 7000 | 1710.32 |
| 2000-03-22 | 7000 | 1720.45 |
| 2000-03-23 | 7000 | 1747.24 |
| 2000-03-24 | 7000 | 1730.5 |
| 2000-03-27 | 7000 | 1775.91 |
| 2000-03-28 | 7000 | 1791.44 |
| 2000-03-29 | 7000 | 1788.81 |
| 2000-03-30 | 7000 | 1810.98 |
| 2000-03-31 | 7000 | 1800.22 |
| 2000-04-03 | 7000 | 1801 |
| 2000-04-04 | 7000 | 1760.69 |
| 2000-04-05 | 7000 | 1771.2 |
| 2000-04-06 | 7000 | 1809.14 |
| 2000-04-07 | 7000 | 1819.9 |
| 2000-04-10 | 7000 | 1826.06 |
| 2000-04-11 | 7000 | 1821.17 |
| 2000-04-12 | 7000 | 1807.33 |
| 2000-04-13 | 7000 | 1831.55 |
| 2000-04-14 | 7000 | 1833.66 |
| 2000-04-17 | 7000 | 1795.06 |
| 2000-04-18 | 7000 | 1813.49 |
| 2000-04-19 | 7000 | 1828.71 |
| 2000-04-20 | 7000 | 1847.03 |
| 2000-04-21 | 7000 | 1841.06 |
| 2000-04-24 | 7000 | 1837.4 |
| 2000-04-25 | 7000 | 1833.47 |
| 2000-04-26 | 7000 | 1832.78 |
| 2000-04-27 | 7000 | 1806.83 |
| 2000-04-28 | 7000 | 1836.32 |
| 2000-05-08 | 7000 | 1836.64 |
| 2000-05-09 | 7000 | 1806.64 |
| 2000-05-10 | 7000 | 1752.69 |
| 2000-05-11 | 7000 | 1724.05 |
| 2000-05-12 | 7000 | 1720.61 |
| 2000-05-15 | 7000 | 1704.59 |
| 2000-05-16 | 7000 | 1724.54 |
| 2000-05-17 | 7000 | 1725.4 |
| 2000-05-18 | 7000 | 1758.54 |
| 2000-05-19 | 7000 | 1777.83 |
| 2000-05-22 | 7000 | 1824.74 |
| 2000-05-23 | 7000 | 1832.08 |
| 2000-05-24 | 7000 | 1855.49 |
| 2000-05-25 | 7000 | 1880.7 |
| 2000-05-26 | 7000 | 1879.62 |
| 2000-05-29 | 7000 | 1898.75 |
| 2000-05-30 | 7000 | 1877.47 |
| 2000-05-31 | 7000 | 1894.55 |
| 2000-06-01 | 7000 | 1903.49 |
| 2000-06-02 | 7000 | 1916.25 |
| 2000-06-05 | 7000 | 1899.09 |
| 2000-06-06 | 7000 | 1925.54 |
| 2000-06-07 | 7000 | 1935.03 |
| 2000-06-08 | 7000 | 1925.21 |
| 2000-06-09 | 7000 | 1900.79 |
| 2000-06-12 | 7000 | 1912.56 |
| 2000-06-13 | 7000 | 1938.67 |
| 2000-06-14 | 7000 | 1932.29 |
| 2000-06-15 | 7000 | 1942.29 |
| 2000-06-16 | 7000 | 1926.94 |
| 2000-06-19 | 7000 | 1915.2 |
| 2000-06-20 | 7000 | 1895.35 |
| 2000-06-21 | 7000 | 1913.13 |
| 2000-06-22 | 7000 | 1913.9 |
| 2000-06-23 | 7000 | 1939.63 |
| 2000-06-26 | 7000 | 1948.64 |
| 2000-06-27 | 7000 | 1942.9 |
| 2000-06-28 | 7000 | 1947.48 |
| 2000-06-29 | 7000 | 1937.26 |
| 2000-06-30 | 7000 | 1928.11 |
| 2000-07-03 | 7000 | 1895.64 |
| 2000-07-04 | 7000 | 1907.93 |
| 2000-07-05 | 7000 | 1909.19 |
| 2000-07-06 | 7000 | 1902.55 |
| 2000-07-07 | 7000 | 1932.79 |
| 2000-07-10 | 7000 | 1948.02 |
| 2000-07-11 | 7000 | 1960.54 |
| 2000-07-12 | 7000 | 1973.37 |
| 2000-07-13 | 7000 | 1979.2 |
| 2000-07-14 | 7000 | 1987.19 |
| 2000-07-17 | 7000 | 1982.48 |
| 2000-07-18 | 7000 | 1993.99 |
| 2000-07-19 | 7000 | 1998.43 |
| 2000-07-20 | 7000 | 1991.56 |
| 2000-07-21 | 7000 | 1996.34 |
| 2000-07-24 | 7000 | 1989.24 |
| 2000-07-25 | 7000 | 1996.58 |
| 2000-07-26 | 7000 | 2012.32 |
| 2000-07-27 | 7000 | 2012.35 |
| 2000-07-28 | 7000 | 2012.79 |
| 2000-07-31 | 7000 | 2023.54 |
| 2000-08-01 | 7000 | 2028.15 |
| 2000-08-02 | 7000 | 2030.68 |
| 2000-08-03 | 7000 | 2036.3 |
| 2000-08-04 | 7000 | 2026.36 |
| 2000-08-07 | 7000 | 2039.42 |
| 2000-08-08 | 7000 | 2049.92 |
| 2000-08-09 | 7000 | 2061.18 |
| 2000-08-10 | 7000 | 2065.94 |
| 2000-08-11 | 7000 | 2062.67 |
| 2000-08-14 | 7000 | 2076.3 |
| 2000-08-15 | 7000 | 2076.82 |
| 2000-08-16 | 7000 | 2083.47 |
| 2000-08-17 | 7000 | 2091.59 |
| 2000-08-18 | 7000 | 2090.18 |
| 2000-08-21 | 7000 | 2108.69 |
| 2000-08-22 | 7000 | 2074.7 |
| 2000-08-23 | 7000 | 2057.92 |
| 2000-08-24 | 7000 | 2071.16 |
| 2000-08-25 | 7000 | 2086.7 |
| 2000-08-28 | 7000 | 2100.59 |
| 2000-08-29 | 7000 | 2105.48 |
| 2000-08-30 | 7000 | 2076.14 |
| 2000-08-31 | 7000 | 2021.2 |
| 2000-09-01 | 7000 | 1999.86 |
| 2000-09-04 | 7000 | 2010.6 |
| 2000-09-05 | 7000 | 1963.79 |
| 2000-09-06 | 7000 | 1925.91 |
| 2000-09-07 | 7000 | 1950.66 |
| 2000-09-08 | 7000 | 1959.31 |
| 2000-09-11 | 7000 | 1972.04 |
| 2000-09-12 | 7000 | 1979.1 |
| 2000-09-13 | 7000 | 1988.94 |
| 2000-09-14 | 7000 | 1979.51 |
| 2000-09-15 | 7000 | 1978.43 |
| 2000-09-18 | 7000 | 1946.75 |
| 2000-09-19 | 7000 | 1903.15 |
| 2000-09-20 | 7000 | 1922.29 |
| 2000-09-21 | 7000 | 1907.31 |
| 2000-09-22 | 7000 | 1891.98 |
| 2000-09-25 | 7000 | 1875.91 |
| 2000-09-26 | 7000 | 1887.83 |
| 2000-09-27 | 7000 | 1914.09 |
| 2000-09-28 | 7000 | 1889.29 |
| 2000-09-29 | 7000 | 1910.16 |
| 2000-10-09 | 7000 | 1915.35 |
| 2000-10-10 | 7000 | 1942.15 |
| 2000-10-11 | 7000 | 1933.73 |
| 2000-10-12 | 7000 | 1926.32 |
| 2000-10-13 | 7000 | 1918.16 |
| 2000-10-16 | 7000 | 1902.81 |
| 2000-10-17 | 7000 | 1909.49 |
| 2000-10-18 | 7000 | 1922.85 |
| 2000-10-19 | 7000 | 1913.42 |
| 2000-10-20 | 7000 | 1947.45 |
| 2000-10-23 | 7000 | 1958.94 |
| 2000-10-24 | 7000 | 1956.34 |
| 2000-10-25 | 7000 | 1977.25 |
| 2000-10-26 | 7000 | 1967.67 |
| 2000-10-27 | 7000 | 1967.4 |
| 2000-10-30 | 7000 | 1973.58 |
| 2000-10-31 | 7000 | 1961.29 |
| 2000-11-01 | 7000 | 1977.36 |
| 2000-11-02 | 7000 | 1984.26 |
| 2000-11-03 | 7000 | 1981.63 |
| 2000-11-06 | 7000 | 1991.17 |

In the following analysis, we use the overnight Shibor and 10-year Treasury bond yield as proxy variables for China’s short-term and long-term interest rates, respectively, and examine the predictive power of the systematic skewness factors on future interest rates.

**5.2.1 Empirical Analysis Based on Bull Markets**

The data processing and control variable selection in Table 7 follow the same procedure as in the full-sample regression. Regarding the prediction of short-term interest rates, the results indicate that during bull market periods, both the *BM2* and *BHS* factors exhibit significant predictive power for the overnight Shibor at the three-month horizon, while the *Cos* factor shows significant predictive ability at the two-month horizon. A significantly negative correlation is observed between future overnight Shibor and all three systematic skewness factors. For long-term interest rate prediction, the findings reveal that during bull markets, the *BM2* and *BHS* factors significantly predict the 10-year Treasury bond yield at the two-month horizon, whereas the *Cos* factor demonstrates predictive power at the one-month horizon. Similarly, a significantly negative correlation is identified between the 10-year Treasury bond yield and each of the three systematic skewness factors.

Our conclusions remain consistent with the empirical results obtained from the full-sample analysis. However, in bull market environments, the systematic skewness factors exhibit predictive power over a longer horizon on short-term interest rates than long-term interest rates. This pattern can be attributed to the fact that during bull markets, monetary authorities pay closer attention to market sentiment in adjusting short-term interest rates to maintain market stability (Bond and Goldstein, 2015), making short-term rates more sensitive to risk perceptions based on systematic skewness. In contrast, long-term interest rates are influenced more strongly by structural macroeconomic factors, which may attenuate the predictive advantage of systematic skewness measures over extended periods. Additionally, the prevailing optimism and higher risk appetite in bullish phases may lead investors to employ shorter-term hedging strategies, further strengthening the persistent predictive power of systematic skewness on short-term rates (Bordalo et al., 2019; Dong et al., 2022).

**Table 7**

Predictive Power of Systematic Skewness Factors on Future Interest Rates (Bull Markets)

| ${Shibor}_{t}=\alpha+\beta_{1}{SysFactor}_{t-n}+\beta_{2}X_{t}+\varepsilon_{t}$ | | | | ${YTB}_{t}=\alpha+\beta_{1}{SysFactor}_{t-n}+\beta_{2}X_{t}+\varepsilon_{t}$ | | | |
| --- | --- | --- | --- | --- | --- | --- | --- |
| $n=1$ | (1) | (2) | (3) | $n=1$ | (1) | (2) | (3) |
| *CONS* | 0.030^***^  (11.76) | 0.027^***^  (11.65) | 0.027^***^  (11.36) | *CONS* | 0.046^***^  (18.78) | 0.035^***^  (18.37) | 0.035^***^  (18.19) |
| $\beta_{Cos}$ | -0.043^**^  (-2.29) |  |  | $\beta_{Cos}$ | -0.027^**^  (-2.03) |  |  |
| $\beta_{BM2}$ |  | -0.065^**^  (-2.56) |  | $\beta_{BM2}$ |  | -0.039^***^  (-2.18) |  |
| $\beta_{BHS}$ |  |  | -0.078^***^  (-2.75) | $\beta_{BHS}$ |  |  | -0.043^***^  (-2.51) |
| *Macroeconomic Variables* | YES | YES | YES | *Macroeconomic Variables* | YES | YES | YES |
| *Adj.R^2^* | 0.59 | 0.60 | 0.62 | *Adj.R^2^* | 0.46 | 0.48 | 0.48 |
| $n=2$ | (1) | (2) | (3) | $n=2$ | (1) | (2) | (3) |
| *CONS* | 0.029^***^  (11.58) | 0.026^***^  (11.61) | 0.025^***^  (11.25) | *CONS* | 0.044^***^  (18.72) | 0.033^***^  (18.47) | 0.033^***^  (18.03) |
| $\beta_{Cos}$ | -0.039^**^  (-1.98) |  |  | $\beta_{Cos}$ | -0.019  (-1.56) |  |  |
| $\beta_{BM2}$ |  | -0.051^**^  (-2.27) |  | $\beta_{BM2}$ |  | -0.031^*^  (-1.75) |  |
| $\beta_{BHS}$ |  |  | -0.062^**^  (-2.53) | $\beta_{BHS}$ |  |  | -0.035^*^  (-1.93) |
| *Macroeconomic Variables* | YES | YES | YES | *Macroeconomic Variables* | YES | YES | YES |
| *Adj.R^2^* | 0.61 | 0.57 | 0.60 | *Adj.R^2^* | 0.45 | 0.49 | 0.51 |
| $n=3$ | (1) | (2) | (3) | $n=3$ | (1) | (2) | (3) |
| *CONS* | 0.028^***^  (11.67) | 0.028^***^  (11.56) | 0.027^***^  (11.14) | *CONS* | 0.042^***^  (18.51) | 0.032^***^  (18.28) | 0.030^***^  (18.12) |
| $\beta_{Cos}$ | -0.026  (-1.02) |  |  | $\beta_{Cos}$ | -0.006  (-0.57) |  |  |
| $\beta_{BM2}$ |  | -0.037^*^  (-1.80) |  | $\beta_{BM2}$ |  | -0.021  (-1.43) |  |
| $\beta_{BHS}$ |  |  | -0.043^**^  (-2.02) | $\beta_{BHS}$ |  |  | -0.020  (-1.35) |
| *Macroeconomic Variables* | YES | YES | YES | *Macroeconomic Variables* | YES | YES | YES |
| *Adj.R^2^* | 0.59 | 0.55 | 0.62 | *Adj.R^2^* | 0.47 | 0.46 | 0.47 |
| $n=4$ | (1) | (2) | (3) | $n=4$ | (1) | (2) | (3) |
| *CONS* | 0.027^***^  (11.39) | 0.025^***^  (11.25) | 0.025^***^  (11.08) | *CONS* | 0.042^***^  (18.63) | 0.031^***^  (18.31) | 0.032^***^  (18.06) |
| $\beta_{Cos}$ | -0.009  (-0.41) |  |  | $\beta_{Cos}$ | -0.007  (-0.79) |  |  |
| $\beta_{BM2}$ |  | -0.013  (-0.62) |  | $\beta_{BM2}$ |  | -0.015  (-1.28) |  |
| $\beta_{BHS}$ |  |  | -0.015  (-0.73) | $\beta_{BHS}$ |  |  | -0.016  (-1.33) |
| *Macroeconomic Variables* | YES | YES | YES | *Macroeconomic Variables* | YES | YES | YES |
| *Adj.R^2^* | 0.57 | 0.56 | 0.59 | *Adj.R^2^* | 0.45 | 0.44 | 0.45 |

*Note:* The significance levels are adjusted using the Newey-West method, with the lag length selected as specified $T^{0.25}\approx4$. ***, ** and * denote significance at the 1%, 5%, and 10% levels, respectively. The values in parentheses are Newey-West adjusted t values.

**5.2.2 Empirical Analysis Based on Bear Markets**

Regarding the prediction of short-term interest rates, the regression results in Table 8 indicate that during bear market phases, both the *BM2* and *BHS* factors exhibit significant predictive power for the overnight Shibor at the two-month horizon, while the *Cos* factor also shows significant predictive ability at the one-month horizon. A significantly negative correlation is observed between the future overnight Shibor and all three systematic skewness factors. With respect to long-term interest rates, the results show that during bear markets, the *BM2* and *BHS* factors demonstrate significant predictive power for the 10-year Treasury bond yield at the three-month horizon, whereas the *Cos* factor exhibits predictive power at the two-month horizon. Similarly, a significantly negative correlation exists between the future 10-year Treasury bond yield and the three systematic skewness factors.

The systematic skewness factors exhibit predictive power over a longer horizon on long-term interest rates than short-term rates - a conclusion that contrasts with the findings observed in bull market conditions. This discrepancy may be attributed to the distinct behaviors and expectations of investors during bear markets. In periods of sustained decline, investors tend to increase their allocation to long-term safe-haven assets to hedge against the risk of falling asset prices. As a result, changes in long-term interest rates primarily reflect current market risk aversion and pessimistic expectations about future economic prospects (Greenwood et al., 2015). In contrast, short-term rates are more susceptible to immediate monetary policies and frequent market fluctuations, resulting in a relatively shorter predictive window. Furthermore, the flight-to-safety phenomenon tends to be more pronounced and structural during bear markets, amplifying the prolonged predictive relationship between skewness factors and long-term yields (Pflueger et al., 2020; Cieslak and Pang, 2021).

**Table 8**

Predictive Power of Systematic Skewness Factors on Future Interest Rates (Bear Markets)

| ${Shibor}_{t}=\alpha+\beta_{1}{SysFactor}_{t-n}+\beta_{2}X_{t}+\varepsilon_{t}$ | | | | ${YTB}_{t}=\alpha+\beta_{1}{SysFactor}_{t-n}+\beta_{2}X_{t}+\varepsilon_{t}$ | | | |
| --- | --- | --- | --- | --- | --- | --- | --- |
| $n=1$ | (1) | (2) | (3) | $n=1$ | (1) | (2) | (3) |
| *CONS* | 0.017^***^  (7.63) | 0.017^***^  (7.39) | 0.017^***^  (7.25) | *CONS* | 0.015^***^  (12.75) | 0.015^***^  (12.62) | 0.015^***^  (12.39) |
| $\beta_{Cos}$ | -0.014^*^  (-1.78) |  |  | $\beta_{Cos}$ | -0.018^**^  (-2.21) |  |  |
| $\beta_{BM2}$ |  | -0.023^**^  (-1.96) |  | $\beta_{BM2}$ |  | -0.027^**^  (-2.57) |  |
| $\beta_{BHS}$ |  |  | -0.029^**^  (-2.19) | $\beta_{BHS}$ |  |  | -0.032^***^  (-2.62) |
| *Macroeconomic Variables* | YES | YES | YES | *Macroeconomic Variables* | YES | YES | YES |
| *Adj.R^2^* | 0.34 | 0.35 | 0.38 | *Adj.R^2^* | 0.35 | 0.37 | 0.40 |
| $n=2$ | (1) | (2) | (3) | $n=2$ | (1) | (2) | (3) |
| *CONS* | 0.017^***^  (7.58) | 0.017^***^  (7.28) | 0.017^***^  (7.12) | *CONS* | 0.015^***^  (12.85) | 0.015^***^  (12.58) | 0.015^***^  (12.45) |
| $\beta_{Cos}$ | -0.010  (-1.37) |  |  | $\beta_{Cos}$ | -0.016^*^  (-1.93) |  |  |
| $\beta_{BM2}$ |  | -0.021^*^  (-1.75) |  | $\beta_{BM2}$ |  | -0.024^**^  (-2.16) |  |
| $\beta_{BHS}$ |  |  | -0.025^*^  (-1.90) | $\beta_{BHS}$ |  |  | -0.028^**^  (-2.24) |
| *Macroeconomic Variables* | YES | YES | YES | *Macroeconomic Variables* | YES | YES | YES |
| *Adj.R^2^* | 0.36 | 0.33 | 0.39 | *Adj.R^2^* | 0.36 | 0.36 | 0.38 |
| $n=3$ | (1) | (2) | (3) | $n=3$ | (1) | (2) | (3) |
| *CONS* | 0.017^***^  (7.45) | 0.017^***^  (7.42) | 0.016^***^  (7.25) | *CONS* | 0.015^***^  (12.95) | 0.015^***^  (12.60) | 0.015^***^  (12.40) |
| $\beta_{Cos}$ | -0.009  (-1.13) |  |  | $\beta_{Cos}$ | -0.013  (-1.42) |  |  |
| $\beta_{BM2}$ |  | -0.017  (-1.34) |  | $\beta_{BM2}$ |  | -0.020^*^  (-1.83) |  |
| $\beta_{BHS}$ |  |  | -0.020  (-1.52) | $\beta_{BHS}$ |  |  | -0.021^*^  (-1.95) |
| *Macroeconomic Variables* | YES | YES | YES | *Macroeconomic Variables* | YES | YES | YES |
| *Adj.R^2^* | 0.34 | 0.35 | 0.37 | *Adj.R^2^* | 0.34 | 0.39 | 0.40 |
| $n=4$ | (1) | (2) | (3) | $n=4$ | (1) | (2) | (3) |
| *CONS* | 0.017^***^  (7.60) | 0.017^***^  (7.55) | 0.016^***^  (7.15) | *CONS* | 0.015^***^  (12.82) | 0.014^***^  (12.42) | 0.014^***^  (12.25) |
| $\beta_{Cos}$ | -0.007  (-0.86) |  |  | $\beta_{Cos}$ | -0.009  (-0.95) |  |  |
| $\beta_{BM2}$ |  | -0.012  (-0.91) |  | $\beta_{BM2}$ |  | -0.015  (-1.21) |  |
| $\beta_{BHS}$ |  |  | -0.012  (-1.09) | $\beta_{BHS}$ |  |  | -0.018  (-1.36) |
| *Macroeconomic Variables* | YES | YES | YES | *Macroeconomic Variables* | YES | YES | YES |
| *Adj.R^2^* | 0.32 | 0.38 | 0.40 | *Adj.R^2^* | 0.36 | 0.37 | 0.39 |

*Note:* The significance levels are adjusted using the Newey-West method, with the lag length selected as specified $T^{0.25}\approx4$. ***, ** and * denote significance at the 1%, 5%, and 10% levels, respectively. The values in parentheses are Newey-West adjusted t values.

The above content has been added in Section 5.2 of the revised manuscript (Pages 13~17 of the revised version) and is highlighted in blue text. **If there are any shortcomings in our revisions regarding this comment, please point them out, and we are willing to make further modifications.**

(5) Your Comment:

***Robustness Checks:*** The paper lacks several standard robustness tests including alternative forecasting horizons beyond 1-4 months, out-of-sample forecasting performance, and alternative specifications for constructing systematic skewness factors (e.g., different portfolio cutoffs than 30/30).

Our revision:

Thank you for this suggestion for revision. We completely agree with your perspective.

In the revised manuscript, we report the predictive ability of the systematic skewness factor for interest rates up to four months ahead across all empirical tables. For forecasting horizons beyond four months, the systematic skewness factor does not demonstrate significant predictive power for future interest rates, and thus such results are not included in the revision.

Additionally, in Sections 5.3 and 5.4, we present out-of-sample forecasting and alternative specifications for constructing systematic skewness factors, respectively. For your convenience, the relevant content (Pages 17~19 of the revised version) is excerpted as follows:

**5.3 Out-of-Sample Forecasting and Comparisons with Benchmark Models**

This section fits the model using historical data from the past five years and forecasts monthly interest rates for the next twelve months. The prediction errors are measured using MSE, RMSE and MAE, with the results shown in Table 9. Whether predicting short-term or long-term interest rates, the prediction errors based on the systematic skewness factors are significantly smaller than those based on the AR model and the extended Taylor rule model proposed by Ma et al. (2025).

**Table 9**

Out-of-Sample Forecasting and Comparisons with AR Model and Extended Taylor Rule Model by Ma et al. (2025)

| Panel A | Out-of-Sample Forecasting of Short-Term Interest Rates | | |
| --- | --- | --- | --- |
| Models | MSE | RMSE | MAE |
| Predicting Shibor Based on *Cos* Factor | 3.15×10^-6^ | 0.0018 | 0.0015 |
| Predicting Shibor Based on *BM2* Factor | 2.89×10^-6^ | 0.0017 | 0.0013 |
| Predicting Shibor Based on *BHS* Factor | 2.34×10^-6^ | 0.0015 | 0.0011 |
| Predicting Shibor Based on AR Model | 4.88×10^-6^ | 0.0028 | 0.0023 |
| Predicting Shibor Based on Extended Taylor Rule Model by Ma et al. (2025) | 7.45×10^-6^ | 0.0047 | 0.0034 |
| Panel B | Out-of-Sample Forecasting of Long-Term Interest Rates | | |
| Models | MSE | RMSE | MAE |
| Predicting YTB Based on *Cos* Factor | 7.13×10^-6^ | 0.0027 | 0.0023 |
| Predicting YTB Based on *BM2* Factor | 6.87×10^-6^ | 0.0026 | 0.0022 |
| Predicting YTB Based on *BHS* Factor | 6.42×10^-6^ | 0.0025 | 0.0021 |
| Predicting YTB Based on AR Model | 7.75×10^-6^ | 0.0028 | 0.0025 |
| Predicting YTB Based on Extended Taylor Rule Model by Ma et al. (2025) | 10.72×10^-6^ | 0.0041 | 0.0035 |

**5.4 Predictive Power Tests Based on Alternative Specifications for Constructing Systematic Skewness Factors**

In this section, the systematic skewness factors are redefined as the return difference between the stocks in the lowest 10% (typically negative) and the highest 10% (typically positive) of systematic skewness. The predictive power of these new systematic skewness factors on future interest rates is examined, and the results are shown in Table 10. For short-term interest rates, all the *Cos*, *BM2* and *BHS* factors have significant predictive power on overnight Shibor over the next 1 to 3 months. For long-term interest rates, the *BHS* factor has significant predictive power on the 10-year Treasury yield over the next 1 to 2 months, whereas the *Cos* and *BM2* factors only show significant predictive power on the 10-year Treasury yield over the next 1 month.

**Table 10**

Predictive Power Tests Based on Alternative Specifications for Constructing Systematic Skewness Factors

| ${Shibor}_{t}=\alpha+\beta_{1}{SysFactor}_{t-n}+\beta_{2}X_{t}+\varepsilon_{t}$ | | | | ${YTB}_{t}=\alpha+\beta_{1}{SysFactor}_{t-n}+\beta_{2}X_{t}+\varepsilon_{t}$ | | | |
| --- | --- | --- | --- | --- | --- | --- | --- |
| $n=1$ | (1) | (2) | (3) | $n=1$ | (1) | (2) | (3) |
| *CONS* | 0.028^***^  (8.57) | 0.027^***^  (8.36) | 0.027^***^  (8.13) | *CONS* | 0.033^***^  (12.90) | 0.033^***^  (12.34) | 0.032^***^  (12.29) |
| $\beta_{Cos}$ | -0.047^**^  (-2.35) |  |  | $\beta_{Cos}$ | -0.038^**^  (-2.08) |  |  |
| $\beta_{BM2}$ |  | -0.055^**^  (-2.50) |  | $\beta_{BM2}$ |  | -0.045^**^  (-2.12) |  |
| $\beta_{BHS}$ |  |  | -0.060^***^  (-2.72) | $\beta_{BHS}$ |  |  | -0.049^**^  (-2.31) |
| *Macroeconomic Variables* | YES | YES | YES | *Macroeconomic Variables* | YES | YES | YES |
| *Adj.R^2^* | 0.47 | 0.50 | 0.52 | *Adj.R^2^* | 0.43 | 0.45 | 0.46 |
| $n=2$ | (1) | (2) | (3) | $n=2$ | (1) | (2) | (3) |
| *CONS* | 0.028^***^  (8.66) | 0.028^***^  (8.49) | 0.027^***^  (8.40) | *CONS* | 0.033^***^  (12.86) | 0.032^***^  (12.35) | 0.032^***^  (12.15) |
| $\beta_{Cos}$ | -0.041^**^  (-2.07) |  |  | $\beta_{Cos}$ | -0.029  (-1.58) |  |  |
| $\beta_{BM2}$ |  | -0.048^**^  (-2.17) |  | $\beta_{BM2}$ |  | -0.036  (-1.62) |  |
| $\beta_{BHS}$ |  |  | -0.052^**^  (-2.43) | $\beta_{BHS}$ |  |  | -0.038^*^  (-1.83) |
| *Macroeconomic Variables* | YES | YES | YES | *Macroeconomic Variables* | YES | YES | YES |
| *Adj.R^2^* | 0.45 | 0.51 | 0.51 | *Adj.R^2^* | 0.41 | 0.44 | 0.44 |
| $n=3$ | (1) | (2) | (3) | $n=3$ | (1) | (2) | (3) |
| *CONS* | 0.028^***^  (8.73) | 0.028^***^  (8.46) | 0.027^***^  (8.29) | *CONS* | 0.033^***^  (12.66) | 0.032^***^  (12.25) | 0.032^***^  (12.14) |
| $\beta_{Cos}$ | -0.036^*^  (-1.78) |  |  | $\beta_{Cos}$ | -0.013  (-0.67) |  |  |
| $\beta_{BM2}$ |  | -0.040^*^  (-1.80) |  | $\beta_{BM2}$ |  | -0.017  (-0.73) |  |
| $\beta_{BHS}$ |  |  | -0.043^**^  (-2.02) | $\beta_{BHS}$ |  |  | -0.019  (-0.95) |
| *Macroeconomic Variables* | YES | YES | YES | *Macroeconomic Variables* | YES | YES | YES |
| *Adj.R^2^* | 0.46 | 0.49 | 0.52 | *Adj.R^2^* | 0.41 | 0.43 | 0.45 |
| $n=4$ | (1) | (2) | (3) | $n=4$ | (1) | (2) | (3) |
| *CONS* | 0.027^***^  (8.44) | 0.027^***^  (8.19) | 0.027^***^  (8.15) | *CONS* | 0.032^***^  (12.79) | 0.032^***^  (12.21) | 0.032^***^  (12.16) |
| $\beta_{Cos}$ | -0.019  (-0.91) |  |  | $\beta_{Cos}$ | -0.008  (-0.49) |  |  |
| $\beta_{BM2}$ |  | -0.023  (-0.96) |  | $\beta_{BM2}$ |  | -0.014  (-0.68) |  |
| $\beta_{BHS}$ |  |  | -0.025  (-1.23) | $\beta_{BHS}$ |  |  | -0.015  (-0.74) |
| *Macroeconomic Variables* | YES | YES | YES | *Macroeconomic Variables* | YES | YES | YES |
| *Adj.R^2^* | 0.48 | 0.50 | 0.51 | *Adj.R^2^* | 0.40 | 0.43 | 0.44 |

*Note:* The significance levels are adjusted using the Newey-West method, with the lag length selected as specified $T^{0.25}\approx4$. ^***^, ^**^ and ^*^ denote significance at the 1%, 5%, and 10% levels, respectively. The values in parentheses are Newey-West adjusted *t* values.

The above content has been added in Sections 5.3 and 5.4 of the revised manuscript (Pages 17~19 of the revised version) and is highlighted in blue text. **If there are any shortcomings in our revisions regarding this comment, please point them out, and we are willing to make further modifications.**

(6) Your Comment:

***Readability and Presentation:*** While the paper is well-organized, several tables (especially Tables 3~6) are excessively dense. The addition of graphical summaries, conceptual diagrams, or flowcharts illustrating relationships between skewness, market sentiment, and interest rates would enhance readability and accessibility for a multidisciplinary audience.

Our revision:

Thank you for this suggestion for revision. We completely agree with your perspective.

In the revised manuscript, we have modified the relevant tables and added Figure 1 to illustrate the relationships among skewness, market sentiment, and interest rates. For your convenience, the corresponding content from the revised manuscript is excerpted as follows:

**Table 3**

Predictive power of Systematic Skewness Factors on Short-Term Interest Rates

| ${Shibor}_{t}=\alpha+\beta_{1}{SysFactor}_{t-n}+\beta_{2}X_{t}+\varepsilon_{t}$ | | | | | | | | | |
| --- | --- | --- | --- | --- | --- | --- | --- | --- | --- |
| $n=1$ | (1) | (2) | (3) | (4) | (5) | (6) | (7) | (8) | (9) |
| *CONS* | 0.027^***^  (20.72) | 0.022^***^  (9.52) | 0.022^***^  (7.93) | 0.027^***^  (20.57) | 0.022^***^  (9.42) | 0.021^***^  (7.86) | 0.027^***^  (20.24) | 0.022^***^  (9.39) | 0.021^***^  (7.81) |
| $\beta_{Cos}$ | -0.042^**^  (-2.38) | -0.034^**^  (-2.13) | -0.030^**^  (-2.06) |  |  |  |  |  |  |
| $\beta_{BM2}$ |  |  |  | -0.047^***^  (-2.61) | -0.039^**^  (-2.40) | -0.036^**^  (-2.32) |  |  |  |
| $\beta_{BHS}$ |  |  |  |  |  |  | -0.052^***^  (-2.90) | -0.041^***^  (-2.67) | -0.038^**^  (-2.59) |
| *Macroeconomic Variables* | NO | YES | YES | NO | YES | YES | NO | YES | YES |
| *Classic Pricing Factors* | NO | NO | YES | NO | NO | YES | NO | NO | YES |
| *Adj.R^2^* | 0.15 | 0.46 | 0.53 | 0.11 | 0.42 | 0.48 | 0.14 | 0.43 | 0.51 |
| $n=2$ | (1) | (2) | (3) | (4) | (5) | (6) | (7) | (8) | (9) |
| *CONS* | 0.027^***^  (20.73) | 0.022^***^  (9.61) | 0.022^***^  (8.01) | 0.027^***^  (20.59) | 0.022^***^  (9.50) | 0.021^***^  (7.97) | 0.027^***^  (20.29) | 0.022^***^  (9.45) | 0.021^***^  (7.89) |
| $\beta_{Cos}$ | -0.033^*^  (-1.91) | -0.026^*^  (-1.70) | -0.024^*^  (-1.67) |  |  |  |  |  |  |
| $\beta_{BM2}$ |  |  |  | -0.041^**^  (-2.40) | -0.034^**^  (-2.15) | -0.033^**^  (-2.09) |  |  |  |
| $\beta_{BHS}$ |  |  |  |  |  |  | -0.045^***^  (-2.67) | -0.036^**^  (-2.46) | -0.035^**^  (-2.38) |
| *Macroeconomic Variables* | NO | YES | YES | NO | YES | YES | NO | YES | YES |
| *Classic Pricing Factors* | NO | NO | YES | NO | NO | YES | NO | NO | YES |
| *Adj.R^2^* | 0.15 | 0.48 | 0.51 | 0.12 | 0.44 | 0.47 | 0.13 | 0.44 | 0.52 |
| $n=3$ | (1) | (2) | (3) | (4) | (5) | (6) | (7) | (8) | (9) |
| *CONS* | 0.027^***^  (20.69) | 0.022^***^  (9.55) | 0.021^***^  (8.03) | 0.027^***^  (20.61) | 0.022^***^  (9.54) | 0.021^***^  (7.90) | 0.027^***^  (20.38) | 0.022^***^  (9.49) | 0.021^***^  (7.68) |
| $\beta_{Cos}$ | -0.025  (-1.53) | -0.017  (-1.35) | -0.017  (-1.31) |  |  |  |  |  |  |
| $\beta_{BM2}$ |  |  |  | -0.036^**^  (-2.13) | -0.031^*^  (-1.94) | -0.030^*^  (-1.89) |  |  |  |
| $\beta_{BHS}$ |  |  |  |  |  |  | -0.039^**^  (-2.41) | -0.032^**^  (-2.22) | -0.030^**^  (-2.14) |
| *Macroeconomic Variables* | NO | YES | YES | NO | YES | YES | NO | YES | YES |
| *Classic Pricing Factors* | NO | NO | YES | NO | NO | YES | NO | NO | YES |
| *Adj.R^2^* | 0.14 | 0.45 | 0.50 | 0.11 | 0.40 | 0.47 | 0.13 | 0.42 | 0.51 |
| $n=4$ | (1) | (2) | (3) | (4) | (5) | (6) | (7) | (8) | (9) |
| *CONS* | 0.027^***^  (20.63) | 0.022^***^  (9.58) | 0.021^***^  (7.99) | 0.027^***^  (20.58) | 0.022^***^  (9.47) | 0.021^***^  (7.96) | 0.027^***^  (20.45) | 0.022^***^  (9.52) | 0.021^***^  (7.73) |
| $\beta_{Cos}$ | -0.011  (-1.06) | -0.06  (-0.70) | -0.05  (-0.67) |  |  |  |  |  |  |
| $\beta_{BM2}$ |  |  |  | -0.022  (-1.56) | -0.19  (-1.30) | -0.14  (-1.15) |  |  |  |
| $\beta_{BHS}$ |  |  |  |  |  |  | -0.021  (-1.48) | -0.13  (-1.09) | -0.10  (-0.87) |
| *Macroeconomic Variables* | NO | YES | YES | NO | YES | YES | NO | YES | YES |
| *Classic Pricing Factors* | NO | NO | YES | NO | NO | YES | NO | NO | YES |
| *Adj.R^2^* | 0.13 | 0.43 | 0.46 | 0.10 | 0.38 | 0.44 | 0.12 | 0.40 | 0.47 |

*Note:* The significance levels are adjusted using the Newey-West method, with the lag length selected as specified $T^{0.25}\approx4$. ^***^, ^**^ and ^*^ denote significance at the 1%, 5%, and 10% levels, respectively. The values in parentheses are Newey-West adjusted *t* values.

**Table 4**

Predictive power of Systematic Skewness Factors on Long-Term Interest Rates

| ${YTB}_{t}=\alpha+\beta_{1}{SysFactor}_{t-n}+\beta_{2}X_{t}+\varepsilon_{t}$ | | | | | | | | | |
| --- | --- | --- | --- | --- | --- | --- | --- | --- | --- |
| $n=1$ | (1) | (2) | (3) | (4) | (5) | (6) | (7) | (8) | (9) |
| *CONS* | 0.036^***^  (30.23) | 0.025^***^  (14.63) | 0.025^***^  (12.75) | 0.036^***^  (30.19) | 0.025^***^  (14.65) | 0.025^***^  (12.66) | 0.036^***^  (29.77) | 0.025^***^  (14.57) | 0.025^***^  (12.67) |
| $\beta_{Cos}$ | -0.028^**^  (-2.19) | -0.023^**^  (-2.00) | -0.022^**^  (-1.96) |  |  |  |  |  |  |
| $\beta_{BM2}$ |  |  |  | -0.030^**^  (-2.28) | -0.025^**^  (-2.00) | -0.024^**^  (-1.97) |  |  |  |
| $\beta_{BHS}$ |  |  |  |  |  |  | -0.039^***^  (-2.92) | -0.033^***^  (-2.66) | -0.032^**^  (-2.57) |
| *Macroeconomic Variables* | NO | YES | YES | NO | YES | YES | NO | YES | YES |
| *Classic Pricing Factors* | NO | NO | YES | NO | NO | YES | NO | NO | YES |
| *Adj.R^2^* | 0.11 | 0.38 | 0.50 | 0.09 | 0.37 | 0.47 | 0.13 | 0.41 | 0.51 |
| $n=2$ | (1) | (2) | (3) | (4) | (5) | (6) | (7) | (8) | (9) |
| *CONS* | 0.036^***^  (30.20) | 0.025^***^  (14.48) | 0.025^***^  (12.59) | 0.036^***^  (30.19) | 0.025^***^  (14.57) | 0.025^***^  (12.75) | 0.036^***^  (29.79) | 0.025^***^  (14.58) | 0.025^***^  (12.60) |
| $\beta_{Cos}$ | -0.025^*^  (-1.87) | -0.022^*^  (-1.74) | -0.020^*^  (-1.70) |  |  |  |  |  |  |
| $\beta_{BM2}$ |  |  |  | -0.029^*^  (-1.95) | -0.024^*^  (-1.83) | -0.023^*^  (-1.78) |  |  |  |
| $\beta_{BHS}$ |  |  |  |  |  |  | -0.038^**^  (-2.59) | -0.033^**^  (-2.37) | -0.031^**^  (-2.35) |
| *Macroeconomic Variables* | NO | YES | YES | NO | YES | YES | NO | YES | YES |
| *Classic Pricing Factors* | NO | NO | YES | NO | NO | YES | NO | NO | YES |
| *Adj.R^2^* | 0.10 | 0.38 | 0.49 | 0.09 | 0.36 | 0.47 | 0.12 | 0.41 | 0.50 |
| $n=3$ | (1) | (2) | (3) | (4) | (5) | (6) | (7) | (8) | (9) |
| *CONS* | 0.036^***^  (30.22) | 0.025^***^  (14.53) | 0.025^***^  (12.70) | 0.036^***^  (30.23) | 0.025^***^  (14.58) | 0.025^***^  (12.69) | 0.036^***^  (29.80) | 0.025^***^  (14.57) | 0.025^***^  (12.28) |
| $\beta_{Cos}$ | -0.022  (-1.46) | -0.018  (-1.28) | -0.017  (-1.22) |  |  |  |  |  |  |
| $\beta_{BM2}$ |  |  |  | -0.024  (-1.61) | -0.020  (-1.45) | -0.019  (-1.36) |  |  |  |
| $\beta_{BHS}$ |  |  |  |  |  |  | -0.035^**^  (-2.23) | -0.030^**^  (-2.06) | -0.028^*^  (-1.93) |
| *Macroeconomic Variables* | NO | YES | YES | NO | YES | YES | NO | YES | YES |
| *Classic Pricing Factors* | NO | NO | YES | NO | NO | YES | NO | NO | YES |
| *Adj.R^2^* | 0.10 | 0.37 | 0.51 | 0.09 | 0.38 | 0.47 | 0.10 | 0.41 | 0.49 |
| $n=4$ | (1) | (2) | (3) | (4) | (5) | (6) | (7) | (8) | (9) |
| *CONS* | 0.036^***^  (30.15) | 0.025^***^  (14.60) | 0.025^***^  (12.68) | 0.036^***^  (30.19) | 0.025^***^  (14.52) | 0.025^***^  (12.74) | 0.036^***^  (29.86) | 0.025^***^  (14.52) | 0.025^***^  (12.23) |
| $\beta_{Cos}$ | -0.017  (-0.84) | -0.012  (-0.75) | -0.012  (-0.71) |  |  |  |  |  |  |
| $\beta_{BM2}$ |  |  |  | -0.020  (-1.01) | -0.014  (-0.96) | -0.013  (-0.94) |  |  |  |
| $\beta_{BHS}$ |  |  |  |  |  |  | -0.035  (-1.52) | -0.030  (-1.46) | -0.028  (-1.43) |
| *Macroeconomic Variables* | NO | YES | YES | NO | YES | YES | NO | YES | YES |
| *Classic Pricing Factors* | NO | NO | YES | NO | NO | YES | NO | NO | YES |
| *Adj.R^2^* | 0.09 | 0.35 | 0.49 | 0.08 | 0.36 | 0.45 | 0.08 | 0.39 | 0.48 |

*Note:* The significance levels are adjusted using the Newey-West method, with the lag length selected as specified $T^{0.25}\approx4$. ^***^, ^**^ and ^*^ denote significance at the 1%, 5%, and 10% levels, respectively. The values in parentheses are Newey-West adjusted *t* values.

**Table 7**

Predictive Power of Systematic Skewness Factors on Future Interest Rates (Bull Markets)

| ${Shibor}_{t}=\alpha+\beta_{1}{SysFactor}_{t-n}+\beta_{2}X_{t}+\varepsilon_{t}$ | | | | ${YTB}_{t}=\alpha+\beta_{1}{SysFactor}_{t-n}+\beta_{2}X_{t}+\varepsilon_{t}$ | | | |
| --- | --- | --- | --- | --- | --- | --- | --- |
| $n=1$ | (1) | (2) | (3) | $n=1$ | (1) | (2) | (3) |
| *CONS* | 0.030^***^  (11.76) | 0.027^***^  (11.65) | 0.027^***^  (11.36) | *CONS* | 0.046^***^  (18.78) | 0.035^***^  (18.37) | 0.035^***^  (18.19) |
| $\beta_{Cos}$ | -0.043^**^  (-2.29) |  |  | $\beta_{Cos}$ | -0.027^**^  (-2.03) |  |  |
| $\beta_{BM2}$ |  | -0.065^**^  (-2.56) |  | $\beta_{BM2}$ |  | -0.039^***^  (-2.18) |  |
| $\beta_{BHS}$ |  |  | -0.078^***^  (-2.75) | $\beta_{BHS}$ |  |  | -0.043^***^  (-2.51) |
| *Macroeconomic Variables* | YES | YES | YES | *Macroeconomic Variables* | YES | YES | YES |
| *Adj.R^2^* | 0.59 | 0.60 | 0.62 | *Adj.R^2^* | 0.46 | 0.48 | 0.48 |
| $n=2$ | (1) | (2) | (3) | $n=2$ | (1) | (2) | (3) |
| *CONS* | 0.029^***^  (11.58) | 0.026^***^  (11.61) | 0.025^***^  (11.25) | *CONS* | 0.044^***^  (18.72) | 0.033^***^  (18.47) | 0.033^***^  (18.03) |
| $\beta_{Cos}$ | -0.039^**^  (-1.98) |  |  | $\beta_{Cos}$ | -0.019  (-1.56) |  |  |
| $\beta_{BM2}$ |  | -0.051^**^  (-2.27) |  | $\beta_{BM2}$ |  | -0.031^*^  (-1.75) |  |
| $\beta_{BHS}$ |  |  | -0.062^**^  (-2.53) | $\beta_{BHS}$ |  |  | -0.035^*^  (-1.93) |
| *Macroeconomic Variables* | YES | YES | YES | *Macroeconomic Variables* | YES | YES | YES |
| *Adj.R^2^* | 0.61 | 0.57 | 0.60 | *Adj.R^2^* | 0.45 | 0.49 | 0.51 |
| $n=3$ | (1) | (2) | (3) | $n=3$ | (1) | (2) | (3) |
| *CONS* | 0.028^***^  (11.67) | 0.028^***^  (11.56) | 0.027^***^  (11.14) | *CONS* | 0.042^***^  (18.51) | 0.032^***^  (18.28) | 0.030^***^  (18.12) |
| $\beta_{Cos}$ | -0.026  (-1.02) |  |  | $\beta_{Cos}$ | -0.006  (-0.57) |  |  |
| $\beta_{BM2}$ |  | -0.037^*^  (-1.80) |  | $\beta_{BM2}$ |  | -0.021  (-1.43) |  |
| $\beta_{BHS}$ |  |  | -0.043^**^  (-2.02) | $\beta_{BHS}$ |  |  | -0.020  (-1.35) |
| *Macroeconomic Variables* | YES | YES | YES | *Macroeconomic Variables* | YES | YES | YES |
| *Adj.R^2^* | 0.59 | 0.55 | 0.62 | *Adj.R^2^* | 0.47 | 0.46 | 0.47 |
| $n=4$ | (1) | (2) | (3) | $n=4$ | (1) | (2) | (3) |
| *CONS* | 0.027^***^  (11.39) | 0.025^***^  (11.25) | 0.025^***^  (11.08) | *CONS* | 0.042^***^  (18.63) | 0.031^***^  (18.31) | 0.032^***^  (18.06) |
| $\beta_{Cos}$ | -0.009  (-0.41) |  |  | $\beta_{Cos}$ | -0.007  (-0.79) |  |  |
| $\beta_{BM2}$ |  | -0.013  (-0.62) |  | $\beta_{BM2}$ |  | -0.015  (-1.28) |  |
| $\beta_{BHS}$ |  |  | -0.015  (-0.73) | $\beta_{BHS}$ |  |  | -0.016  (-1.33) |
| *Macroeconomic Variables* | YES | YES | YES | *Macroeconomic Variables* | YES | YES | YES |
| *Adj.R^2^* | 0.57 | 0.56 | 0.59 | *Adj.R^2^* | 0.45 | 0.44 | 0.45 |

*Note:* The significance levels are adjusted using the Newey-West method, with the lag length selected as specified $T^{0.25}\approx4$. ^***^, ^**^ and ^*^ denote significance at the 1%, 5%, and 10% levels, respectively. The values in parentheses are Newey-West adjusted *t* values.

**Table 8**

Predictive Power of Systematic Skewness Factors on Future Interest Rates (Bear Markets)

| ${Shibor}_{t}=\alpha+\beta_{1}{SysFactor}_{t-n}+\beta_{2}X_{t}+\varepsilon_{t}$ | | | | ${YTB}_{t}=\alpha+\beta_{1}{SysFactor}_{t-n}+\beta_{2}X_{t}+\varepsilon_{t}$ | | | |
| --- | --- | --- | --- | --- | --- | --- | --- |
| $n=1$ | (1) | (2) | (3) | $n=1$ | (1) | (2) | (3) |
| *CONS* | 0.017^***^  (7.63) | 0.017^***^  (7.39) | 0.017^***^  (7.25) | *CONS* | 0.015^***^  (12.75) | 0.015^***^  (12.62) | 0.015^***^  (12.39) |
| $\beta_{Cos}$ | -0.014^*^  (-1.78) |  |  | $\beta_{Cos}$ | -0.018^**^  (-2.21) |  |  |
| $\beta_{BM2}$ |  | -0.023^**^  (-1.96) |  | $\beta_{BM2}$ |  | -0.027^**^  (-2.57) |  |
| $\beta_{BHS}$ |  |  | -0.029^**^  (-2.19) | $\beta_{BHS}$ |  |  | -0.032^***^  (-2.62) |
| *Macroeconomic Variables* | YES | YES | YES | *Macroeconomic Variables* | YES | YES | YES |
| *Adj.R^2^* | 0.34 | 0.35 | 0.38 | *Adj.R^2^* | 0.35 | 0.37 | 0.40 |
| $n=2$ | (1) | (2) | (3) | $n=2$ | (1) | (2) | (3) |
| *CONS* | 0.017^***^  (7.58) | 0.017^***^  (7.28) | 0.017^***^  (7.12) | *CONS* | 0.015^***^  (12.85) | 0.015^***^  (12.58) | 0.015^***^  (12.45) |
| $\beta_{Cos}$ | -0.010  (-1.37) |  |  | $\beta_{Cos}$ | -0.016^*^  (-1.93) |  |  |
| $\beta_{BM2}$ |  | -0.021^*^  (-1.75) |  | $\beta_{BM2}$ |  | -0.024^**^  (-2.16) |  |
| $\beta_{BHS}$ |  |  | -0.025^*^  (-1.90) | $\beta_{BHS}$ |  |  | -0.028^**^  (-2.24) |
| *Macroeconomic Variables* | YES | YES | YES | *Macroeconomic Variables* | YES | YES | YES |
| *Adj.R^2^* | 0.36 | 0.33 | 0.39 | *Adj.R^2^* | 0.36 | 0.36 | 0.38 |
| $n=3$ | (1) | (2) | (3) | $n=3$ | (1) | (2) | (3) |
| *CONS* | 0.017^***^  (7.45) | 0.017^***^  (7.42) | 0.016^***^  (7.25) | *CONS* | 0.015^***^  (12.95) | 0.015^***^  (12.60) | 0.015^***^  (12.40) |
| $\beta_{Cos}$ | -0.009  (-1.13) |  |  | $\beta_{Cos}$ | -0.013  (-1.42) |  |  |
| $\beta_{BM2}$ |  | -0.017  (-1.34) |  | $\beta_{BM2}$ |  | -0.020^*^  (-1.83) |  |
| $\beta_{BHS}$ |  |  | -0.020  (-1.52) | $\beta_{BHS}$ |  |  | -0.021^*^  (-1.95) |
| *Macroeconomic Variables* | YES | YES | YES | *Macroeconomic Variables* | YES | YES | YES |
| *Adj.R^2^* | 0.34 | 0.35 | 0.37 | *Adj.R^2^* | 0.34 | 0.39 | 0.40 |
| $n=4$ | (1) | (2) | (3) | $n=4$ | (1) | (2) | (3) |
| *CONS* | 0.017^***^  (7.60) | 0.017^***^  (7.55) | 0.016^***^  (7.15) | *CONS* | 0.015^***^  (12.82) | 0.014^***^  (12.42) | 0.014^***^  (12.25) |
| $\beta_{Cos}$ | -0.007  (-0.86) |  |  | $\beta_{Cos}$ | -0.009  (-0.95) |  |  |
| $\beta_{BM2}$ |  | -0.012  (-0.91) |  | $\beta_{BM2}$ |  | -0.015  (-1.21) |  |
| $\beta_{BHS}$ |  |  | -0.012  (-1.09) | $\beta_{BHS}$ |  |  | -0.018  (-1.36) |
| *Macroeconomic Variables* | YES | YES | YES | *Macroeconomic Variables* | YES | YES | YES |
| *Adj.R^2^* | 0.32 | 0.38 | 0.40 | *Adj.R^2^* | 0.36 | 0.37 | 0.39 |

*Note:* The significance levels are adjusted using the Newey-West method, with the lag length selected as specified $T^{0.25}\approx4$. ^***^, ^**^ and ^*^ denote significance at the 1%, 5%, and 10% levels, respectively. The values in parentheses are Newey-West adjusted *t* values.

**Figure 1**

The relationship among hedging demands, systematic skewness factors and future interest rates

Increased market fear and risk aversion

Increased market optimism and risk appetite

Rising investor preference for stocks with positive systematic skewness

Declining investor preference for stocks with positive systematic skewness

The value of the systematic skewness factor increases

The value of the systematic skewness factor decreases

Predicting future interest rates

**If there are any shortcomings in our revisions regarding this comment, please point them out, and we are willing to make further modifications.**

(7) Your Comment:

***Policy and Practical Implications:*** The conclusion section could more explicitly articulate the implications for monetary policy, portfolio management, and risk assessment. For instance, how might systematic skewness serve as a market-based indicator of future policy shifts or investor sentiment during liquidity tightening cycles?

Our revision:

Thank you for this suggestion for revision. We completely agree with your perspective.

In the revised manuscript, we have emphasized in multiple sections that when investors’ hedging demand increases, they tend to purchase more stocks with positive systematic skewness, leading to the overvaluation of these stocks, a decline in future returns, and a corresponding rise in the value of the systematic skewness factor. This signal is transmitted through monetary policy and significantly influences future interest rates. Moreover, for both short-term and long-term interest rates, prediction errors based on systematic skewness factors are consistently lower than those from an AR model and the extended Taylor‑rule model proposed by Ma et al. (2025). Systematic skewness can serve as an asymmetric pricing signal in the market for extreme interest rate risks. Its increase often reflects heightened investor anxiety over liquidity tightening, thereby offering central banks a forward-looking sentiment monitoring window independent of traditional economic indicators.

**If there are any shortcomings in our revisions regarding this comment, please point them out, and we are willing to make further modifications.**
